# The association between combustible/electronic cigarette use and stroke based on national health and nutrition examination survey

**DOI:** 10.1186/s12889-023-15371-x

**Published:** 2023-04-14

**Authors:** Jing Shi, Lijun Xiong, Jun Guo, Yan Yang

**Affiliations:** 1 Health Management Center, Tongji Medical College, The Central Hospital of Wuhan, Huazhong University of Science and Technology, 430014 Wuhan, China; 2 Department of Neurology, Tongji Medical College, The Central Hospital of Wuhan, Huazhong University of Science and Technology, 430014 Wuhan, China

**Keywords:** Stroke, Combustible cigarette, Electronic cigarette, Smoking, Clinical prediction model.

## Abstract

**Aims:**

This study aims to analyze the association between combustible/electronic cigarettes and the risk of stroke.

**Methods:**

We obtained data from the 2017–2018 National Health and Nutrition Examination Survey (NHANES). The stroke history and combustible/electronic cigarette use were acquired by questionnaires. Considering the sole or dual use of combustible cigarettes and electronic cigarettes (e-cigarettes), we divided all the individuals into four subgroups, including nonsmokers (reference group), sole combustible cigarette, sole e-cigarette, and dual use of both combustible cigarettes and e-cigarettes. We performed multivariable logistic regression to determine the association between cigarette use with the prevalence of stroke. We used odds ratios (ORs) with 95% confidence intervals (CIs) to show the effect size. Finally, we developed a prediction model to evaluate the risk of stroke for individuals with combustible or electronic cigarette use based on a random forest model.

**Results:**

We included a total of 4022 participants in the study. The median age was 55, and 48.3% of the participants were males. When we adjusted for age, gender, education attainment, race, total-to-HDL cholesterol (< 5.9 or ≥ 5.9), diabetes, hypertension, and alcohol consumption, the groups of sole e-cigarette use, sole combustible cigarette use, and dual use of combustible and electronic cigarettes were significantly associated with the prevalence of stroke with ORs (with 95%CI) of 2.07 (1.04–3.81), 2.36 (1.52–3.59), 2.34 (1.44–3.68), respectively. In the testing set, the AUC was 0.74 (95%CI = 0.65–0.84), sensitivity was 0.68, and specificity was 0.75.

**Conclusion:**

Sole e-cigarettes and dual use of e-cigarettes with combustible cigarettes might increase the risk of stroke.

## Introduction

It is estimated that about 795,000 people experience stroke each year in the US, which causes serious health threats and has become the fourth leading cause of death in the US [1]. Accumulating evidence has well demonstrated that smoking could significantly increase the risk of ischemic stroke in a dose-dependent manner [2–5]. In the US, it is estimated that about 17,800 stroke deaths were attributed to smoking annually, which accounted for about 12-14% of all stroke death [6].

Studies on traditional combustible cigarettes were primarily conducted in a population of older age, whereas recent studies extended to younger individuals showed consistent results [7, 8]. Smoking contributes to the development of stroke with both short-term and long-term effects [9]. Even smoking one cigarette immediately increases heart rate, blood pressure, and cardiac index, contributing to thrombus generation in atherosclerotic arteries [10]. Besides the immediate effect, long-term cigarette exposure (both active and passive) accelerates the development and progress of atherosclerosis [11].

E-cigarettes, also known as vaping, are battery-powered electronic nicotine delivery systems containing the solution of water and chemicals. Different from traditional combustible cigarettes, e-cigarettes are battery-powered electronic nicotine delivery systems containing the solution of water and many harmful constituents, such as nicotine, diacetyl, fine ultrafine particles, heavy metals, and carbonyl compounds [12]. Modern electronic cigarettes were initially introduced to the international market more than a decade ago as an alternative tool for quitting smoking. In the past decade, the landscape of e-cigarettes has been evolving and expanding, and many e-cigarette devices have been introduced to the market in various shapes and sizes. The latest generation of e-cigarettes is the fourth generation, known as “pod” devices. Nicotinic salt-based formulations with extremely high nicotine concentrations can be used in the latest e-cigarette without unpalatable sensory experiences [13]. The use of e-cigarettes has substantially increased among both smokers and nonsmokers in the US, particularly in children and young adults [13–15]. The prevalence of sole e-cigarette use grew to about 1.9 million in 2016, and 17.7% were daily users [14]. Therefore, special attention has recently been raised to the escalating health crisis caused by electronic cigarette (e-cigarette) use.

Despite the increasing popularity of e-cigarettes, it remains unclear whether e-cigarette use elevates the risk of stroke or other cardiovascular diseases [16]. A recent cross-sectional study on 465,594 participants from the 2016 behavioral risk factor surveillance system suggested that current e-cigarette use was positively associated with increased stroke risk compared with those who never use e-cigarettes [17]. Also, another study reported that e-cigarette users showed a 1.15-fold risk of stroke than combustible cigarette users [18]. Contractively, a recent meta-analysis showed no significant association between e-cigarette use and the risk of stroke with a pooled odd ratio (OR) of 1.13 and 95% confidence interval (CI) of 0.99–1.29 compared with nonsmokers [19]. Therefore, further research should be performed to evaluate the effect of combustible/electronic cigarette use on the risk of stroke, which would improve the management strategy for e-cigarette use.

Therefore, we investigated the association between combustible/electronic cigarette use and the prevalence of stroke using the national wide population data.

## Methods

### Study population

We obtained data from the National Health and Nutrition Examination Survey (NHANES) database, a survey program designed to evaluate the health and nutrition status in the United States. The National Center for Health Statistics and the Centers for Disease Control and Prevention conducted the NHANES survey from the early 1960s. Since 1999, the survey has become a continuous program conducted every two years, and each survey visited about 5000 individuals. Multiple health and nutrition interview and examination data types were collected, including demographics, dietary, examination, laboratory, and questionnaire data subtypes.

We included individuals from the NHANES 2017–2018 survey. Smoking status, body mass index, medical conditions, alcohol consumption, and standard biochemistry profiles were analyzed in this study. Individuals aged below 30 or above 80 were excluded from the study. National Health and Nutrition Examination Survey Ethics Review Board approved the 2017–2018 survey (Continuation of Protocol #2011-17 and Protocol #2018-01) [20]. Informed consent was obtained from all subjects.

### Outcomes

NHANES database provides self-reported personal interviews on health conditions and medical history, which is coded as the MCQ questionnaire Sect. [21]. All participants were asked the following question: “Has a doctor or other health professional ever told you that you had a stroke?” (Questionnaire code: MCQ160f). Participants who answered “Yes” was defined as patients with stroke. The strategy to identify patients with stroke was consistent with previous studies [22–24]. Participants with unknown stroke status were excluded from the study.

### Combustible or electronic cigarette use

In the questionnaire survey, the Smoking Cigarette use dataset (coded as SMQ questionnaire section) collects recodes on cigarette use, current use, past 30-day prevalence, amount, and other related smoking details. Participants were asked whether they had smoked at least 100 cigarettes in their entire life (Questionnaire code: SMQ020) and their current smoking habits (Questionnaire code: SMQ040). Participants were asked whether they used e-cigarettes (battery-powered devices containing liquid nicotine without producing smoke) even once (Questionnaire code: SMQ900). Combustible cigarette use was defined as smoking at least 100 cigarettes in entire life and presently smoking cigarettes every day or some days. E-cigarette use was defined as using e-cigarettes even once [25]. Based on the above questions, we divided the participants into four subgroups as follows: (1) nonsmokers (not combustible cigarettes smokers or e-cigarette smokers), (2) sole combustible cigarette use (combustible cigarettes smokers but not e-cigarette smokers), (3) sole electronic cigarette use (e-cigarette smokers but not combustible cigarettes smokers), (4) dual use of both combustible and electronic cigarettes. The classification followed a previous study on electronic and combustible cigarette use [25].

### Covariates

Age, gender (male, female), race (non-Hispanic White, non-Hispanic Black, Mexican American, other Hispanic, and other race), education attainment (below high school, high school, and above high school), weight, body mass index, creatinine, triglycerides, cholesterol, fasting plasm glucose, and alcohol consumption were obtained. The estimated glomerular filtration rate (eGFR) was calculated by the Cockroft and Gault formula, and patients with eGFR < 60 mL/min/1.73m^2^ was defined with chronic kidney disease. Patients with self-reported hypertension or taking hypertension prescription was classified as hypertension. Patients with diabetes were defined as those who reported diabetes diagnosis, fasting plasma glucose above 126 mg/dL, or HbA1c ≥ 6.5%.

### Statistical analysis

We described the participants’ characteristics using median with interquartile range (continuous variables) or percentages (categorical variables). One-way analysis of variance test, Kruskal-Wallis test, or chi-square test was used to compare the characteristics between groups as appropriate. We performed multivariable logistic regression to determine the association between cigarette use and stroke prevalence. In the adjusted model, we adjusted for age (continuous variable), gender (male or female), education attainment (below high school, high school, or above high school), race (non-Hispanic White, non-Hispanic Black, Mexican American, other Hispanic, and other race), total-to-HDL cholesterol (< 5.9 or ≥ 5.9), diabetes (no or yes), hypertension (no or yes), and alcohol consumption (no or yes). We used ORs with 95% CIs to show the effect size. Moreover, we developed a prediction model to evaluate the risk of stroke for individuals with combustible or electronic cigarette use based on a random forest model. P value < 0.05 was considered statistically significant. We used R software. R software (version 4.1) was used to perform the statistical analyses.

## Results

### Participants’ characteristics

We included a total of 4022 participants in the study. The median age was 55, and 48.3% of the participants were males. Participants with no cigarette use, e-cigarette only, combustible cigarette only, and both combustible and electronic cigarette account for 74.9%, 5.8%, 9.5%, and 9.8%, respectively. Compared with the control group, participants in the stroke group were of significantly higher age (63 vs. 55), fasting plasma glucose (97.0 vs. 95.0), and HbA1c (5.9% vs. 5.7%). More patients with diabetes and hypertension were in the stroke group. No significant difference was observed in gender, body mass index, triglycerides, and alcohol consumption. Participants’ characteristics are shown in Table 1.

**Table 1:** Participants’ characteristics

	All participants (n = 4022)	Stroke group (n = 201)	Normal group (n = 3821)	P value
Age (year)	55.0 (43.0, 65.0)	63.0 (55.0, 70.0)	55.0 (42.0, 64.0)	< 0.001
Gender (Male, %)	48.3	50.20	48.20	0.612
Race (%)				< 0.001
Non-Hispanic White	32.0	34.8	31.9	
Non-Hispanic Black	24.3	37.8	23.6	
Mexican American	13.5	8.0	13.7	
Other Hispanic	9.9	4.5	10.2	
Other races	20.3	14.9	20.6	
Education attainment (%)				< 0.001
Below high school	20.6	25.4	20.4	
High School	22.8	31.3	22.3	
Above high school	56.6	43.3	57.3	
Body mass index (kg/m^2^)	29.0 (25.4, 34.0)	30.3 (25.6, 35.1)	28.9 (25.4, 33.9)	0.253
Diabetes (Yes, %)	26.1	45.3	25.0	< 0.001
Fasting plasma glucose (mg/dL)	95.0 (88.0, 105.0)	97.0 (89.0, 118.0)	95.0 (88.0, 105.0)	0.029
Hemoglobin A1c (%)	5.7 (5.4, 6.1)	5.9 (5.5, 6.6)	5.7 (5.4, 6.1)	< 0.001
Hypertension (Yes, %)	42.3	76.6	40.5	< 0.001
Triglycerides (mg/dL)	123.0 (88.0, 178.0)	129.5 (91.2, 178.8)	123.0 (88.0, 178.0)	0.268
Total-to-HDL cholesterol	3.7 (2.9, 4.5)	3.5 (2.7, 4.3)	3.7 (3.0, 4.6)	0.010
eGFR (mL/min/1.73m^2^)	104.6 (81.2, 133.4)	80.4 (60.5, 105.5)	105.6 (82.4, 134.6)	< 0.001
Cigarette use (%)				< 0.001
Nonsmokers	74.9	63.7	75.5	
Sole e-cigarette use	5.8	6.0	5.8	
Sole combustible cigarette use	9.5	16.9	9.2	
Dual use	9.8	13.4	9.6	
Alcohol consumption (Yes, %)	83.8	83.6	83.8	1.000

eGFR: Estimated glomerular filtration rate

### The association between cigarette use and stroke

Table 2 shows the logistic regression analysis of the association between combustible/electronic cigarette use and stroke. In the crude logistic regression model, sole combustible cigarette use (OR = 2.19, 95% CI = 1.46–3.21) and dual use of combustible and electronic cigarettes (OR = 1.66, 95% CI = 1.06–2.51) were associated with an increased risk of stroke when setting nonsmokers as reference. However, we observed no significant effect of sole e-cigarette use (OR = 1.23, 95% CI = 0.64–2.17, P = 0.50). In the adjusted model, the groups of sole e-cigarette use, sole combustible cigarettes use, and dual use were all significantly associated with the prevalence of stroke with ORs (with 95%CI) of 2.07 (1.04–3.81), 2.36 (1.52–3.59), and 2.34 (1.44–3.68), respectively.

**Table 2:** The association between cigarette use and the prevalence of stroke

	Crude model		Adjusted model	
	Odds ratio	*P*-value	Odds ratio	*P*-value
Nonsmokers	Reference		Reference	
Sole e-cigarette	1.23 (0.64–2.17)	0.505	2.07 (1.04–3.81)	0.027
Sole combustible cigarette	2.19 (1.46–3.21)	< 0.001	2.36 (1.52–3.59)	< 0.001
Dual use	1.66 (1.06–2.51)	0.02	2.34 (1.44–3.68)	< 0.001

Adjusted model: We adjusted for age, gender, education attainment, race, total-to-HDL cholesterol, diabetes, hypertension, and alcohol consumption

### A prediction model to evaluate the stroke risk for individuals with cigarette use

**Fig. 1:**
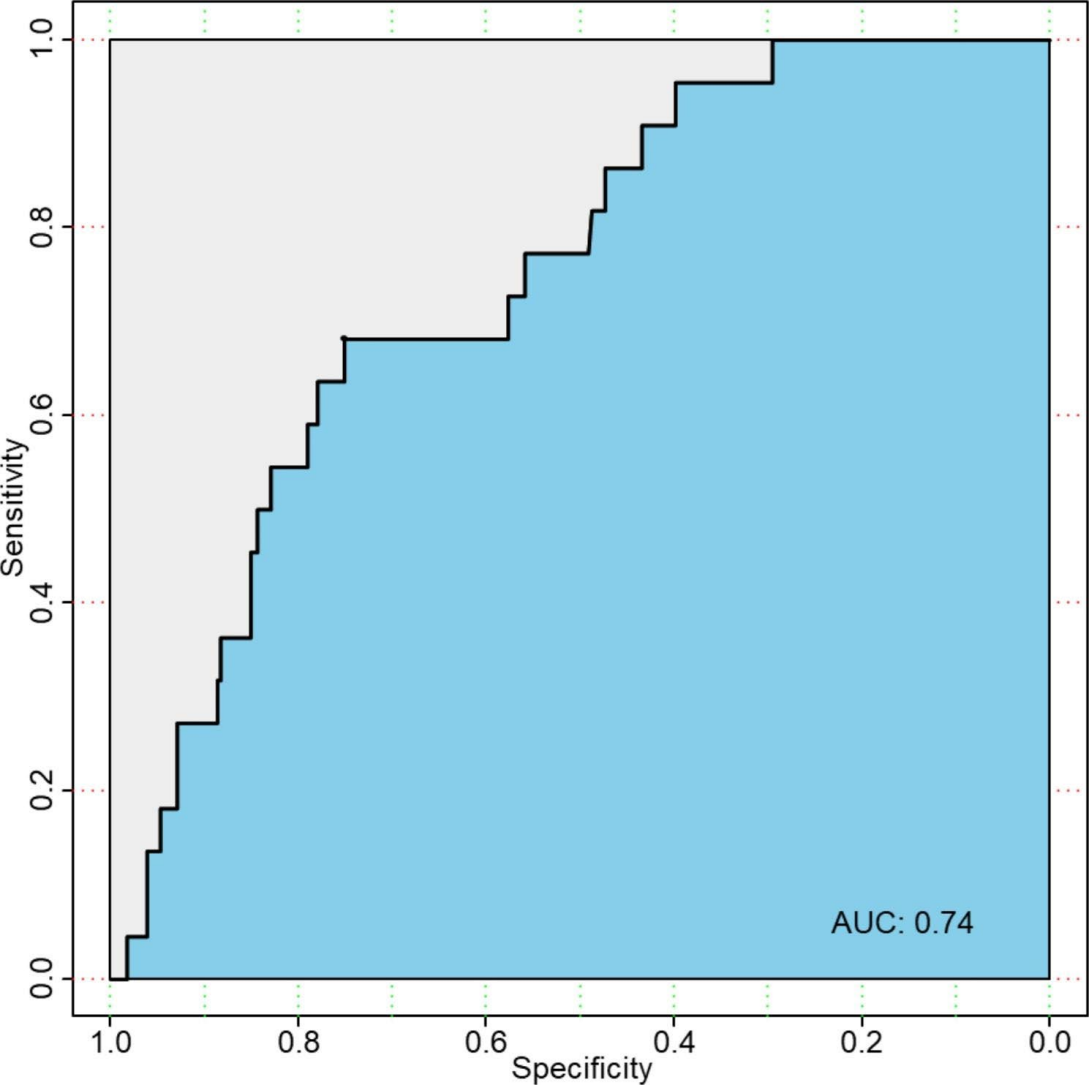
The receive operate curve of the prediction model

We used cigarette use, age [26], gender [27], race [28], education attainment [29], diabetes [30], hypertension[31], total-to-HDL cholesterol [32], and alcohol consumption [33] to develop a random forest-based prediction model, which aims to evaluate the stroke risk for individuals with cigarette use. In the testing set, the AUC was 0.74 (95%CI = 0.65–0.84), sensitivity was 0.68, and specificity was 0.75. Figure 1 shows the ROC curve of the prediction model.

## Discussion

In our study, we investigated the association between combustible/electronic cigarettes and the prevalence of stroke based on 4022 participants from the NHANES study. After adjusting for age, gender, education attainment, race, total-to-HDL cholesterol, diabetes, hypertension, and alcohol consumption, the group of dual use of combustible and electronic cigarette was significantly associated with the increased prevalence of stroke with an OR 2.34 (95%CI = 1.44–3.68) compared with the nonsmokers. Consistent with our finding, a cross-sectional survey of 161,529 participants aged between 18 and 44 years showed a 2.91-fold risk of stroke in those with dual use than nonsmokers [34].

Importantly, our results showed that when compared with nonsmokers, sole e-cigarette use was significantly associated with a 2.07-fold risk of stroke (95%CI = 1.04–3.81). The adverse effect of e-cigarette use on stroke was also reported in other studies [17, 34]. Bricknell and colleagues analyzed the association between e-cigarette use and stroke using the 2016 behavioral risk factor surveillance system, and their results indicated that current e-cigarette use was positively associated with increased stroke [17]. Modern e-cigarettes were initially used as an effective alternative tool to help smokers quit smoking, which was considered less harmful compared to traditional combustible cigarettes. The current guidelines for the primary prevention of stroke recommend quitting combustible cigarette smoking for the active smoker in many manners [7]. However, it should be noticed that e-cigarettes could generate harmful volatile organic compounds at high temperatures, such as acetaldehyde and formaldehyde, apart from toxic metals and propylene glycol [12, 35, 36]. An in vitro study on the ischemic stroke model showed that e-cigarette vaping could result in glucose deprivation in the neurovascular unit, which potentially leads to elevated stroke risk and enhanced ischemic brain injury [37]. Also, Patel et al. [18] reported that e-cigarette users showed a higher risk of stroke than combustible cigarette users (adjusted OR = 1.15, 95% CI = 1.15–1.16). Moreover, evidence suggested that e-cigarette use might result in additional cardiovascular health risks for combustible cigarette users due to the ingredients of e-cigarettes. For example, a study by Parekh et al. [34] showed that the dual use of both combustible and electronic cigarette could induce a 1.83-fold risk than sole combustible cigarette use.

Differently, the study on 161,529 young adults aged 18–44 years revealed that sole e-cigarette use showed no significant association with the risk of stroke compared with nonsmokers [34]. The contractive results might be caused by the younger age population, which was associated with low health risk and higher insurance enrollment. A recent meta-analysis based on cross-sectional studies showed that e-cigarette use did not significantly increase the risk of stroke with a pooled OR of 1.13 (95% CI: 0.99–1.29) compared with nonsmokers [19]. However, it should be noted that the meta-analysis showed high heterogeneity with an I^2^ of 45.9%, and the difference in population age might result in the contract results. These results indicate that further studies on the cardiovascular effect of e-cigarettes should consider population age as an important covariate in the study design and statistical analysis.

Together with previous studies [17, 34], our results suggested that e-cigarettes might not be a safe alternative to combustible cigarette. Despite that e-cigarettes were initially labeled as an effective option for smoking cessation, concerns were raised about the remaining nicotine dependence and toxicity in e-cigarettes, especially the young adults [38]. Current findings indicate that physicians and public health agencies should consider the use of e-cigarettes with caution. Recently, FDA has initiated the regulation of all e-cigarettes, which aims to protect children and young adults from unapproved e-cigarette devices [13]. Still, the role of e-cigarette use or the dual use of e-cigarettes with combustible cigarettes in the development of stroke requires more evidence and there lacks sufficient evidence to improve the better management of combustible/electronic cigarette use.

Although the large sample size improved the statistical power and reliability of the results, some limitations should be mentioned. First of all, this study is a cross-sectional design, and it is unclear whether stroke events occurred before or after the e-cigarette use initiation. Therefore, the results from this study are insufficient to establish the causality between combustible/electronic cigarette use and stroke. More evidence from prospective studies should be subsequently performed to further evaluate the health risk of e-cigarettes. Second, the adverse effect of e-cigarettes on cardiovascular health is dose-dependent. The quantitative analysis should be further performed in the following analysis [39]. Third, the prevalence of stroke and cigarette use information was obtained from questionnaires. Although data from questionnaires have been widely used in clinical research [23, 24], it is concerning that the questionnaire-based data might result in bias. Moreover, this study was conducted on the US population, which makes it uncertain whether the conclusion can be extended to different countries and areas considering the different races and lifestyles.

## Conclusion

Our study showed that combustible/electronic cigarette use might elevate the risk of stroke. Although preliminary, the results showed concerns about the health safety of e-cigarettes. Current data supported that longitudinal studies should be performed to further evaluate the adverse cardiovascular effect caused by e-cigarettes and dual use.

## Data Availability

The data of this study can be found on the NHANES database (https://wwwn.cdc.gov/nchs/nhanes/Default.aspx).
